# Supplementary material for: The genome sequence of the Sycamore-seed Pygmy moth, Ectoedemia decentella (Herrich-Schäffer, 1855) van Nieukerken, 1986
Source: Wellcome Open Res. 2025 Apr 8;10:182. [Version 1] doi: 10.12688/wellcomeopenres.23973.1 (PMC12485360; doi:10.12688/wellcomeopenres.23973.1)
Supplement: Peer review history

# Reviewer report: Reviewer response for version 1

**DOI:** 10.21956/wellcomeopenres.26449.r122115

**Published:** 2025-05-09

**Authors:** Sivasankaran Kuppusamy

**Affiliations:** 1 Loyola College, Chennai, India

The chromosome-level genome of *Ectoedemia decentella* (Herrich-Schaffer, 1855) van Nieukerken, 1986 was sequenced and assembled by the authors. They have appropriate methods for DNA isolation and they have used advanced software tools for the genome assembly and annotation.

 Authors have assembled 418.14 Mb of total genome in size. They scaffolded into 31 chromosome pseudomolecules in assembly.

**Comments on the manuscript**

 The first sentence of the background can start “ *Ectoedemia decentella *(Herrich-Schaffer, 1855) van Nieukerken, 1986 is a leaf-mining….”

 Throughout the manuscript, the authors consistently used the genus name *Ectoedemia decentella* in its full form. If possible, use the full genus name at first, and later to shorten it to *E. decentella*.

 Authors have produced almost 100 Gb data. Even though they haven’t observed and listed the number of protein-coding genes, number of non-coding genes and number of gene transcripts in the table. Any reason?

 The research article was well prepared and the manuscript meets the necessary scientific standard and is suitable for indexing"

Are sufficient details of methods and materials provided to allow replication by others?

Yes

Is the rationale for creating the dataset(s) clearly described?

Yes

Are the datasets clearly presented in a useable and accessible format?

Yes

Are the protocols appropriate and is the work technically sound?

Yes

Reviewer Expertise:

Phyogenetic analysis of Noctuoidea moths using mitogenome sequence

I confirm that I have read this submission and believe that I have an appropriate level of expertise to confirm that it is of an acceptable scientific standard.

# Reviewer report: Reviewer response for version 1

**DOI:** 10.21956/wellcomeopenres.26449.r122119

**Published:** 2025-05-08

**Authors:** Panagiotis Ioannidis

**Affiliations:** 1 Foundation for Research & Technology - Hellas, Crete, Greece

This paper describes the sequencing and assembly of a lepidopteran species following the standard methodology.  Moreover, the assembly is publicly available and free to use by anyone who's interested.

 My impression is that, with 83.7% complete BUSCOs, the assembly is somewhat incomplete. Previous insect (lepidopteran, even) assemblies have >>90% complete BUSCOs. If the authors would like to comment on the possible causes for this, that would be great.

 Other than this, I have a minor correction to suggest; in the "Assembly quality metrics" section the authors say "...identified 83.7% of the expected gene set...". I'd suggest they change this to "...identified 83.7% of the BUSCOs...", because a "gene set" is something else.

Are sufficient details of methods and materials provided to allow replication by others?

Yes

Is the rationale for creating the dataset(s) clearly described?

Yes

Are the datasets clearly presented in a useable and accessible format?

Yes

Are the protocols appropriate and is the work technically sound?

Yes

Reviewer Expertise:

Insect genomics, bioinformatics

I confirm that I have read this submission and believe that I have an appropriate level of expertise to confirm that it is of an acceptable scientific standard.
